# Sociocultural factors in eating disorder. A comprehensive review

**DOI:** 10.1192/j.eurpsy.2025.1413

**Published:** 2025-08-26

**Authors:** G. Strada, B. Serván, L. Reyes, I. López, M. Díaz

**Affiliations:** 1 Hospital Universitario Clínico San Carlos, Madrid, Spain

## Abstract

**Introduction:**

Eating disorders, such as anorexa nervosa, bulimia nervosa and binge eating disorder, are complex conditions that affect both a person’s physical and mental health. Although biological and psychological factors have been extensively studied, sociocultural factors play a fundamental role in the developmental and maintenance of these disorders.

**Objectives:**

This poster explores how peer pressure, beauty standards, the influence of the media and social media, along with aspects such as gender, social class and culture, contribute to the emergence of eating disorders, especially among teenagers and young women.

**Methods:**

Literature review

**Results:**

Patterns are identified that show a significant correlation between the idealization of thinness and increased body dissatisfaction. The impact of patriarchy, diet culture and social media consumption are discussed, and how these influences are amplified in different sociocultural contexts. Additionally, differences in the prevalence of eating disorders by gender and ethnicity are discussed, as well as implications for treatment prevention.

**Conclusions:**

Intervention strategies that address sociocultural factors and promote a more inclusive and healthy view of the body are needed, with the aim of reducing the impact of these factors on the appearance of eating disorders. The research highlights the need for public policies that regulate media content and promote educational programs on physical and mental health.

**Disclosure of Interest:**

None Declared

